# Diversity and Distribution of Deep-Sea Shrimps in the Ross Sea Region of Antarctica

**DOI:** 10.1371/journal.pone.0103195

**Published:** 2014-07-22

**Authors:** Zeenatul Basher, David A. Bowden, Mark J. Costello

**Affiliations:** 1 Institute of Marine Science, The University of Auckland, Auckland, New Zealand; 2 Coasts and Oceans Centre, National Institute of Water and Atmospheric Research (NIWA), Wellington, New Zealand; Institute of Marine Research, Norway

## Abstract

Although decapod crustaceans are widespread in the oceans, only Natantia (shrimps) are common in the Antarctic. Because remoteness, depth and ice cover restrict sampling in the South Ocean, species distribution modelling is a useful tool for evaluating distributions. We used physical specimen and towed camera data to describe the diversity and distribution of shrimps in the Ross Sea region of Antarctica. Eight shrimp species were recorded: *Chorismus antarcticus; Notocrangon antarcticus; Nematocarcinus lanceopes; Dendrobranchiata*; *Pasiphaea scotiae*; *Pasiphaea* cf. *ledoyeri*; *Petalidium* sp., and a new species of *Lebbeus*. For the two most common species, *N. antarcticus* and *N. lanceopes*, we used maximum entropy modelling, based on records of 60 specimens and over 1130 observations across 23 sites in depths from 269 m to 3433 m, to predict distributions in relation to environmental variables. Two independent sets of environmental data layers at 0.05° and 0.5° resolution respectively, showed how spatial resolution affected the model. *Chorismus antarcticus* and *N. antarcticus* were found only on the continental shelf and upper slopes, while *N. lanceopes, Lebbeus* n. sp., *Dendrobranchiata, Petalidium* sp., *Pasiphaea* cf. *ledoyeri,* and *Pasiphaea scotiae* were found on the slopes, seamounts and abyssal plain. The environmental variables that contributed most to models for *N. antarcticus* were depth, chlorophyll-*a* concentration, temperature, and salinity, and for *N. lanceopes* were depth, ice concentration, seabed slope/rugosity, and temperature. The relative ranking, but not the composition of these variables changed in models using different spatial resolutions, and the predicted extent of suitable habitat was smaller in models using the finer-scale environmental layers. Our modelling indicated that shrimps were widespread throughout the Ross Sea region and were thus likely to play important functional role in the ecosystem, and that the spatial resolution of data needs to be considered both in the use of species distribution models.

## Introduction

Natant decapod crustacea (shrimp and prawns) are ubiquitous in the world’s oceans and shallow seas, including the Antarctic, where other decapod taxa are largely absent [1], [2], [3], [4], [5], [6]. As they are predominantly benthic particulate feeders and predators, they can be important in processing of material at the seabed [7], [8], [9]. Studies by Arntz & Gorny [10] and Gutt *et al.* [11] using underwater photography, have described species composition, distributions, and habitats of three shrimp species in the Weddell Sea but no similar studies have been conducted for the Ross Sea. The benthic fauna of the Ross Sea continental shelf has been relatively well-studied, particularly in coastal regions, and shares many taxa with other sectors of the Antarctic [12], [13], [14], [15], but deeper benthic habitats of the shelf edge, slope and abyssal depths remain little-sampled [16], [17], [18]. Brandt *et al.* [18] have highlighted the high rate of discovery for new species from the deep Southern Ocean, where up to 86% of isopod crustacean species were new to science, and argued that priority should be given to identifying the spatial distribution and abundance of key species in each trophic group across the region. Decapods are key species in the functioning of marine ecosystems, world-wide, as predators, scavengers, detritivores, and prey [19], [20]. In the Ross Sea region, information on the distributions and population densities of shrimps is necessary for producing ecosystem models which will improve understanding of trophic interactions and inform environmental management [21].

Because sampling in the Antarctic is restricted by remoteness, intense seasonality, and sea-ice, species distribution models (SDM) may provide a useful tool for estimating species’ occurrences from limited field sample data. The basic assumption of SDM is that the fundamental niche of a species, defined by physiological and ecological tolerances, is the primary driver of its realized distribution [22], [23]. Few SDM studies have focused on marine invertebrates, yet these groups have several attributes that make them well suited to species distribution modelling. Restricted availability of marine data [24], and a limited number of high quality species occurrence records were considered as obstacles behind the application of SDM in the ocean [25], [26]. In recent years, as more sophisticated modelling algorithms have become available, the potential to model species’ distributions across un-sampled marine regions is now realistic. In this study, we used MaxEnt, a machine-learning algorithm based on the principle of maximum entropy [27], which has been shown to have superior performance among presence-only algorithms [28] for species distribution modelling [29], [30].

Marine environmental datasets available for use in SDM have varying spatial resolutions and are frequently provided in different file formats, making the data assembly a time-consuming aspect of SDM studies [25]. Studies in the terrestrial domain have found that coarser spatial resolution resulted in reduced accuracy of predicted area although overall in SDM performance was not affected [31], [32], [33], [34]. However, the effect of spatial resolution on the relative influence of environmental variable on species distributions has not been assessed. The availability of several environmental datasets for the present study area, each with different spatial resolution provided an opportunity to investigate the effect of spatial resolution on the influence of environmental variables and the accuracy of the predicted area in the marine environment for the first time. In this study we used sample data on shrimp distribution in the Ross Sea, Antarctica, with historical records of occurrence, and two sets of environmental variables to (a) explore the diversity and distribution of shrimps in the Ross Sea region, (b) model distributions of suitable habitat for two common species, and (c) investigate the effect of using datasets with differing spatial resolutions on model predictions in the marine environment.

## Methods

### Study area

**Figure 1:**
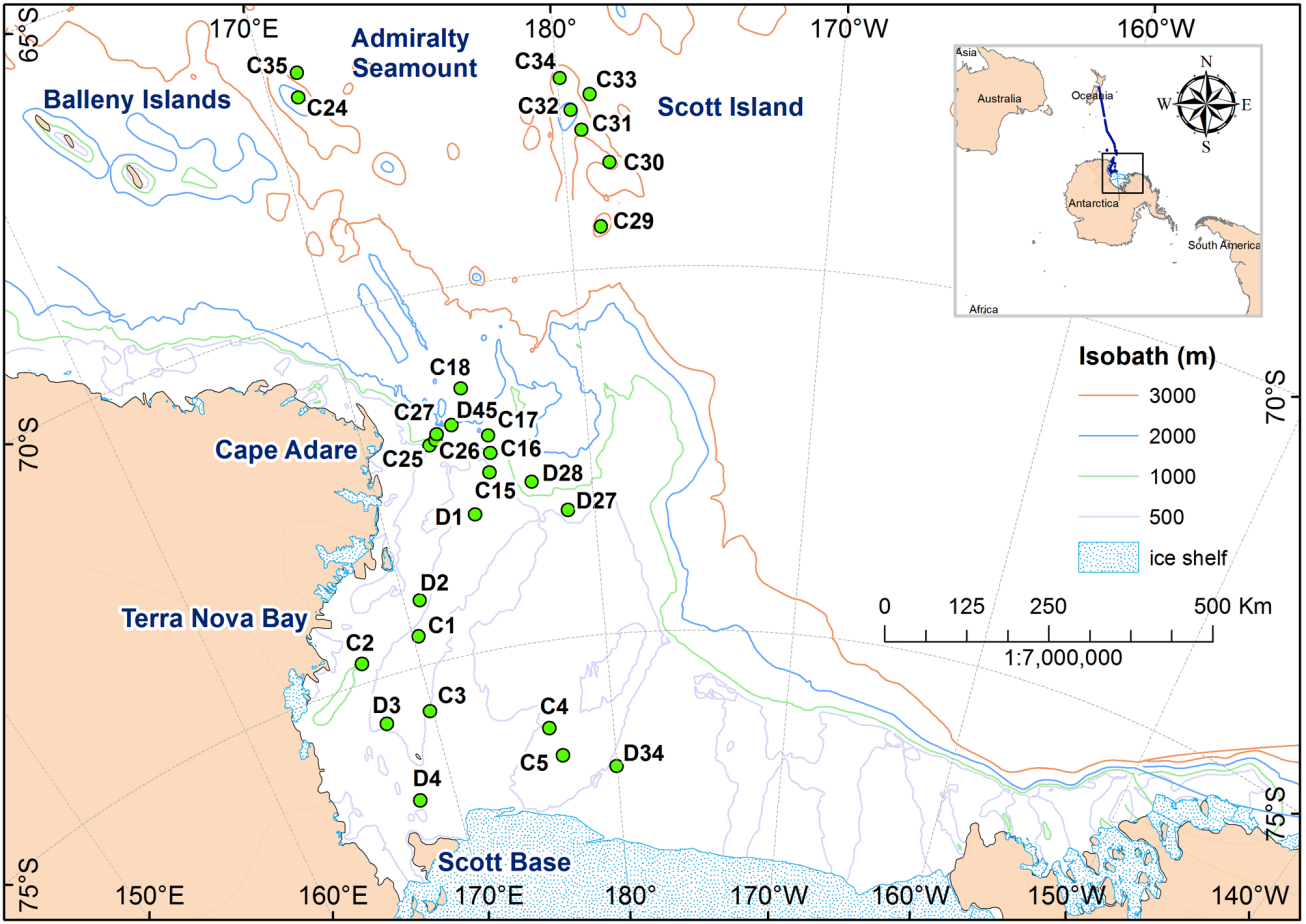
Map of Ross Sea region showing sampling sites of the New Zealand International Polar Year–Census of Antarctic Marine Life (NZ IPY-CAML) voyage TAN0802.

Our study area was bounded by 65°S, 150°E, 140°W, and the Ross ice shelf in the south. It included the entire Ross Sea continental shelf area, the Balleny Islands, and Scott and Admiralty seamounts (Figure 1). The mean depth of the Ross Sea continental shelf is about 500 m, although depth varies widely between deep troughs and shallow banks, and the area free of glaciers and permanent ice shelves is ca. 433,061 km^2^ (delineated by 800 m isobath and the Ross Ice Shelf).

### Sampling

The Ross Sea region is not a protected area in the Antarctica and is under the jurisdiction of Ross Sea Dependency of New Zealand. The study did not involve collecting any endangered or protected species. Samples were collected from 12 February to 11 March 2008 during New Zealand’s 2008 International Polar Year–Census of Antarctic Marine Life voyage (IPY-CAML, RV *Tangaroa*, TAN0802) at 10 sites on the Ross Sea continental shelf, 10 sites on the northern continental slope, 3 sites on the abyssal plain (>3000 m depth), and 5 seamounts to the north (Figure 1 and Table 1). At each site, at least one, and up to 7, 1 hour deployments of a towed camera system with high definition digital video and still image cameras were made [35]. The camera array (NIWA’s Deep Towed Imaging System, DTIS) was held ca. 2.5 m above the seabed and towed at 0.25–0.5 ms^−1^. In total, 55 camera transects were run. The seabed position was recorded in real time using an ultra short baseline (USBL) acoustic transponder system (Simrad HPR 410). Camera transects at each site were followed by physical sampling gear including a beam trawl (4 m width, 25 mm mesh), a large demersal fish trawl (25 m wing spread, 40 mm mesh), and two types of epibenthic sled; a fine mesh (1 m width, 0.5 mm mesh) sled used on flat, smooth seabeds [36], and a coarse mesh (1 m width, 25 mm mesh) sled used on seamounts. In addition, a fine-mesh midwater trawl, was used following acoustic surveys. It had a circular mouth opening of about 12 m diameter and a cod end mesh of 10 mm and was generally towed for 20–30 min at 3–4 knots. All shrimp specimens collected by trawls and sleds were preserved (except in large hauls where representative 2–5 specimens/station were preserved) in 99% ethanol and were identified to species level.

**Table 1:** Number of shrimp specimens collected or observed at each station, site and region.

Region	Site	Station	Co-ordinates	Gear	Depth (Mean)	*Chorismus antarcticus*	*Dendrobranchiata*	*Lebbeus* n. sp.	*Nematocarcinus lanceopes*	*Notocrangon antarcticus*	*Pasiphaea* cf. *ledoyeri*	*Pasiphaea scotiae*	*Petalidium* sp.
Abyssal	C30	186	−68.52, −178.3	DTIS	3227				5				
		189	−68.56, −178.3	BT	3207							2	
	C33	228	−67.61, −178.8	DTIS	3366				3				
		230	−67.61, −178.8	BT	3480							1	
	C35	285	−66.73, 171.18	DTIS	2711				7				
Seamount	C24	276	−67.01, 171.07	DTIS	695				12				
		278	−67.01, 171.07	DTIS	771				7				
		280	−67.16, 171.16	DTIS	587			30					
		281	−67.16, 171.16	EBS	604			2^a^					
		293	−66.99, 171.08	MWT	1032							2	1
		294	−66.94, 170.99	DTIS	2055				5				
		295	−66.93, 170.82	DTIS	553			2					
		301	−67.13, 171.16	DTIS	1024				7				
		302	−67.13, 171.14	EBS	947				7^a^				
		303	−67.12, 171.09	FT	743			8			5^a^		
		304	−67.16, 171.18	DTIS	642			11	9				
		305	−67.16, 171.17	EBS	634			2^a^					
		307	−67.17, 171.12	EBS	616			4^a^					
		309	−67.12, 170.89	EBS	738			1	5^a^				
		312	−67.00, 170.69	MWT	1078							1	
	C31	194	−68.13, −179.3	MOC	110				**2** ^ab^				
		199	−68.10, −179.3	EBS	634			1					
		201	−68.09, −179.2	EBS	730				2				
		202	−68.07, −179.3	DTIS	1138				34				
		203	−68.08, −179.2	EBS	895				1				
		205	−68.11, −179.2	DTIS	864				54				
		206	−68.12, −179.2	EBS	876				10				
		207	−68.14, −179.2	DTIS	1191				46				
		210	−68.11, −179.3	EBS	662				3				
		211	−68.10, −179.2	FT	867				65^a^		3^a^		
	C32	218	−67.72, −179.7	EBS	1173				2				
		219	−67.78, −179.7	DTIS	1180				8				
		220	−67.78, −179.7	EBS	1189				2				
		224	−67.73, −179.6	EBS	841				1				
	C33	227	−67.60, −178.8	MWT	1000							1	
	C34	237	−67.40, −179.8	EBS	1540				4				
		244	−67.38, −179.8	DTIS	718				224				
		245	−67.38, −179.8	EBS	660				7				
		250	−67.37, 133.82	DTIS	1440				39				
		251	−67.38, 179.98	EBS	1496				3				
		255	−67.34, −179.9	DTIS	1027				42				
		256	−67.34, −179.9	EBS	1183			1	1				
	C35	283	−66.94, 171.33	MOC	800				1^b^				
		284	−66.79, 171.24	MWT	1004								1
Shelf	C1	26	−74.58, 170.24	FT	285	2^a^							
		31	−74.59, 170.27	BT	283	11^a^				8			
	C2	40	−74.73, 167.01	DTIS	898					34			
		41	−74.72, 167.01	FT	923					41^a^			
		43	−74.77, 167.05	HBS	800					1			
		46	−74.73, 167.06	BT	865					4			
	C3	55	−75.63, 169.78	DTIS	530	4				79			
		56	−75.63, 169.85	FT	528					18^a^			
		61	−75.62, 169.80	BT	521					9^a^			
	C4	93	−76.19, 176.29	DTIS	450	41				44			
		94	−76.19, 176.29	FT	447	1				33^a^			
		100	−76.20, 176.24	BT	449	1				10^a^			
	C5	80	−76.60, 176.77	DTIS	368	26				65			
		81	−76.59, 176.82	FT	367					17			
		82	−76.59, 176.88	HBS	363					8			
		84	−76.60, 176.80	BT	360					7			
	D2	22	−74.11, 170.79	FT	636					1			
	D3	65	−75.62, 167.33	DTIS	269	4				4			
		66	−75.62, 167.32	FT	477					10^a^			
	D34	76	−76.83, −179.9	DTIS	664	1				3			
		77	−76.83, −179.9	FT	664	1				7^a^			
	D4	69	−76.80, 167.87	DTIS	706					23			
		70	−76.77, 167.83	FT	731					11^a^			
Slope	C17	130	−72.08, 175.55	DTIS	1565				159				
		133	−72.09, 175.57	FT	1577				50		3^a^		
		139	−72.08, 175.55	BT	1620				5				
	C18	169	−71.38, 174.73	DTIS	2213				60				
		171	−71.38, 174.73	FT	2282				5				
	C25	158	−72.07, 172.92	MOC	450				1^b^				
	C27	142	−71.98, 173.39	MWT	1005								1
	D28	108	−72.82, 177.13	DTIS	1369				110				
		109	−72.80, 177.19	FT	1413				20^a^			1	
	D45	166	−71.84, 174.00	DTIS	1917				44				
		167	−71.85, 174.03	FT	1972		1		479^a^				
Upper Slope	C26	150	−72.02, 173.17	DTIS	795					1			
	D27	105	−73.25, 178.72	DTIS	775				3				
		106	−73.24, 178.72	FT	757					2			

a Total number of specimens caught in the haul, not all specimens preserved for further analysis;

b Larval specimen collected from pelagic MOCNESS (See Wiebe *et al.* [67] for gear specification and Gallego *et al.* [91] for specimen details) deployment.

**Gear type:** FT = Fish Trawl; BT = Beam Trawl; MOC = MOCNESS; MWT = Mid-water Trawl; HBS = Hyperbenthic Sled; EBS = Epibenthic Sled; DTIS = Deep Towed Imaging System.

Post-voyage analyses of video transects were run using Ocean Floor Observation Protocol (OFOP; www.ofop-by-sams.eu) software. Raw USBL transponder positions were first smoothed using a running mean and splined with associated metadata (e.g., time, depth, heading, vehicle altitude) to yield corrected seabed tracks with position coordinates and metadata values at 1-s intervals. The digital video files were then synchronised with the corrected position data to enable re-running of transects in the laboratory with full video playback control and precise spatial and temporal logging of events. All shrimps on all transects were recorded and identified as close to species level as possible, using the high-resolution still images to confirm identities.

### Environmental variables

**Figure 2:**
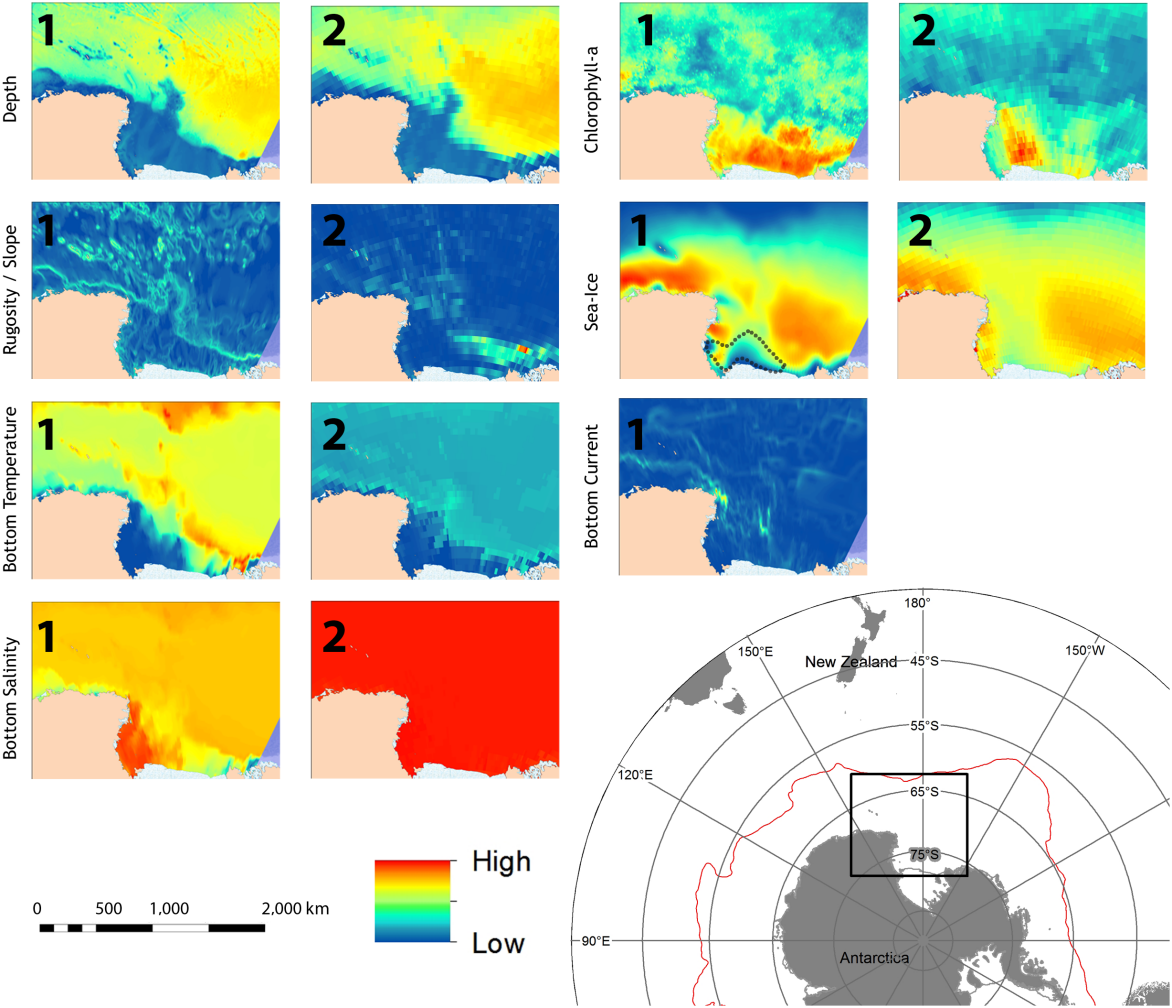
Environmental layers used for modelling. Numbers denote respective environmental datasets. The location of the Ross Sea polynya is marked with dash in the sea ice layer.

We compiled environmental variables from two different sources (referred to hereafter as SET 1 and SET 2), each with different spatial resolution (Table 2, 3 and Figure 2). We selected variables that were likely to be ecologically relevant to benthic distributions: depth; seabed slope or rugosity; bottom temperature; ice concentration (proportion of the year with >85% ice cover in SET 1, annual mean in SET 2); chlorophyll-*a* concentration (mean summer in SET 1, mean annual concentration in SET 2) and for SET 1 only, bottom current speed.

**Table 2:** Details and sources of environmental variables used for modelling.

Set	Data Layer	Description	Reference
1	Depth	Water depth taken from GEBCO_O8 Digital Atlas	IOC et al. [92]
	Rugosity	The rugosity layer is an approximation to true rugosity defined as the actual area of seabed divided by the area projected onto an equipotential (horizontal) plane.	Burrough & McDonnell [93]
	Chlorophyll-*a*	Mean SeaWiFS surface Chl-a in Summer (Dec-Feb), natural log averaged between 1997–2007	Hooker et al. [94], NASA [95]
	Temperature	Bottom temperature from HIGEM 1.1 Model	Shaffrey et al. [96] & Rickard et al. [37]
	Salinity	Bottom salinity from HIGEM 1.1 Model	Shaffrey et al. [96] & Rickard et al. [37]
	Ice Concentration	Fraction of the year for which a given pixel was covered with >85% from Nmbus-7 & DMSP satellites dated 1979/80 to 2006/07 seasons.	U.S. National Snow and Ice Data Centre ([97], Updated 2007)
	Current	Current speed (*speed*) by combining the modelled meridional and zonal velocities from HiGEM 1.1 model	Shaffrey et al. [96] & Rickard et al. [37]
2	Depth	Mean ETOPO 2 min bathymetry (negative) elevation in 30 min cell	Smith and Sandwell [98]
	Slope	Slope derived from depth layer using ArcGIS Spatial Analyst	This study
	Chlorophyll-*a*	Proportion of annual primary production in a cell in mgC·m−^2^·day ^−1^.	Bouvet *et al.* [99], Hoepffner *et al.* [100], Longhurst *et al.* [101]
	Temperature	Mean annual sea bottom temperature as derived from WOA 2001 Bottom Source Information for all coastal and oceanic cells. Coverage 1990–1999	Stephens et al. [102]
	Salinity	Mean annual bottom salinity in Practical Salinity Scale (PPS), as derived from WOA 2001 Bottom Source Information for all coastal and oceanic cells. Coverage 1990–1999	Boyer et al. [103]
	Ice Concentration	Mean annual ice cover in percent as derived from the National Snow and Ice Data Centre (1979–2002)	U.S. National Snow and Ice Data Centre [97], Updated 2006

SET 1 was at 0.05°and SET 2 at 0.5° latitude-longitude.

**Table 3:** Summary statistics for the environmental variables in each dataset used in models (SD = Standard deviation, SE = Standard error, CV = Coefficient of variance).

	Variable	Unit	Min	Max	Mean	SD	SE	CV
SET 1	Depth	m	3.25	6044.70	2461.74	1557.52	2.23	0.63
(0.05°)	Rugosity	% (0–1)	0	0.70	0.08	0.05	0	0.67
	Temperature	Degree C	−1.79	0.73	−0.46	0.67	0.001	−1.48
	Salinity	ppt	34.13	34.85	34.66	0.07	0	0
	Chlorophyll-*a* (Summer mean)	ln (mgC·m−^2^·day^−1^)	−0.94	0.91	−0.27	0.37	0	−1.36
	Ice Concentration	% (0–1)	0	0.78	0.27	0.23	0	0.85
	Current	cm s^−1^	0.05	57.00	2.02	2.20	0.003	1.09
SET 2	Depth	m	0	5304.00	2559.47	1621.75	16.17	0.63
(0.50°)	Slope	Degree	0	4.52	0.17	0.31	0.003	1.79
	Temperature	Degree C	−2.01	1.57	0.03	0.71	0.007	26.45
	Salinity	ppt	33.72	34.94	30.99	10.68	0.105	0.34
	Chlorophyll-*a* (Annual mean)	mgC·m−^2^·day ^−1^ cell ^−1^	0	2.50	0.62	0.40	0.004	0.65
	Ice Concentration	% (0–1)	0	1.00	0.42	0.37	0.003	0.87

All variables except ice and Chlorophyll-a concentration were for the seabed or near seabed.

SET 1 had a spatial resolution of 0.05° longitude and 0.05° latitude [37], [38], representing approximately 5.5 km by 2 km at areas between 67°S and 68°S, and consisted of 7 variables derived from satellite observations and modelled climatologies. SET 2 had a spatial resolution of 0.5° and consisted of 6 variables obtained from AquaMaps [39] (Table 2).

All datasets were received in raw csv format, and interpolated to raster layers at the respective spatial resolutions using the “Spatial Analyst” extension in ArcGIS 10. Inverse distance weighted (IDW) multivariate interpolation [40], [41] was used in the ArcGIS Spatial Analyst extension with default setting and smoothing (p = 2) option to assign the final interpolated cell value in the generated raster layers. Chlorophyll-*a* was transformed to natural log to improve normality in SET 1 (Table 3). Raster layers were converted to ASCII grid with WGS84 Antarctic Polar Stereographic projection. The finer resolution dataset (SET 1) had almost 45 times more grid cells across the study region than the coarser resolution dataset (ca. 450,000 vs 10,000).

There were missing values in some layers in SET 2 but not in SET 1. During raster interpolation, these ‘no data’ pixels were assigned average values of 12 surrounding (ocean) cells using ArcGIS raster calculator. The “Band Collection Statistics” multivariate toolset function [42] of Spatial Analyst was used to calculate Pearson’s correlation coefficient between the variables in each dataset (Table S1). Correlation coefficients over ±0.7 were considered significant [43], [44] and are known to affect model prediction capability [45], [46].

### Modelling fitting procedure

Using all occurrence data from TAN0802 physical and photographic samples, we modelled the two most commonly-occurring shrimp species, *Notocrangon antarcticus* and *Nematocarcinus lanceopes*, using MaxEnt version 3.3.3e (http://www.cs.princeton.edu/~schapire/maxent/), with each of the two sets of environmental variables as predictors in consecutive runs for each species (Table 2 and Figure 2). Our occurrence records were distributed over 160,000 km^2^, which is sufficiently spatially segregated to reduce the probability of spatial-correlation between observations [30], [47]. MaxEnt is flexible with respect to the types of variables used and the form of their relationship to a species’ presence (e.g. linear, nonparametric, etc.). A review comparing 16 models of >200 taxa found that machine-learning methods including MaxEnt consistently outperformed traditional linear methods [28] and that presence-only models were preferable because limited sampling may mean that apparent absences may not be true. We selected the ‘Auto features’ function for model fit in MaxEnt, which automatically applies the feature or features estimated to be appropriate for the particular sample size of occurrence records [48]. As the number of records varied depending the resolution of the datasets in this study, only linear, quadratic and hinge features (See [49] for definitions) were utilized for model fitting.

MaxEnt models were generated using 100 bootstrap replicates run with the ‘random seed’ option turned on. The ‘Remove duplicate presence records’ feature was enabled to exclude duplicate records that fell within individual pixels of background environment layers on each dataset and the occurrence records were split into 75% for training and 25% for testing for bootstrap replications. The Maximum number of background points (randomly selected in each replication) was increased to 100,000 instead of the default value of 10,000 because of our large-scale mapping objective. Maximum iterations were also increased to 1000 allowing enough time for model convergence. As suggested by Phillips & Dudik [48] the default regularisation value was used because it results in better performance of evaluation data for presence-only datasets. We also used the settings ‘fade by clamping’ option to minimize unreliable extrapolation into areas with environmental conditions that were not encountered during model training. The relative contributions of variables were calculated in the MaxEnt models in training steps where the algorithm keeps track of how much each environmental variable contributes to fitting the model and adjusts the overall gain to calculate contributions of individual variables.

### Model evaluation

Various test statistics are available to test the ability of models to discriminate suitable versus unsuitable habitat [50], [51]. Several studies have highlighted issues with using only one statistic to evaluate model performance [52], [53]. Options for model validation include: (1) internal validation, or cross-validation in which the data are partitioned randomly into ‘training’ and ‘test’ sets, thus creating quasi-independent data for model evaluation [32], [50] using the Area Under the receiver operating Curve (AUC) [48], [49], [54] criterion; (2) omission rates [55], [56]; (3) low presence threshold (LPT) [57], and (4) completely independent datasets [28], [58], [59], [60], [61]. We validated our models using all four of these methods.

AUC measures the quality of a ranking of sites [62]. Use of AUC analysis with presence-only evaluation datasets has been justified for the presence versus random classification problem [63]. AUC is measured on a scale of 0–1, where 1 indicates no errors of omission or commission, 0.5 indicates no better than random selection, and 0.9 indicates that there is a 90% chance that predicted habitat suitability for a randomly drawn species presence will be higher than that of randomly drawn absence [61], [63], [64]. MaxEnt provides AUC values based on the evaluation localities used in each model run. In this study, mean AUC values calculated from 100 bootstrap models were used to measure model performance. MaxEnt’s built-in Jack-knife validation method was also used as an independent estimate of each variable’s contribution to overall model performance allowing comparison with AUC values for each variable.

The threshold-dependent intrinsic (based on training data) or extrinsic (based on test data) *omission rate*, is the fraction of the known presence localities that fall into pixels not predicted as suitable for the species. A low omission rate is indicative of a good model [55]. High-quality models should show zero or low omission of evaluation localities, or at least predict evaluation localities statistically better than random.

LPT sets the lowest threshold value of the prediction for any of the presence localities in the training dataset (measured on a scale of 0–1) [57]. This yields a binary prediction that includes all pixels that are at least as suitable (according to the model) as those where the species was known to be present (in the training dataset). These threshold values generally vary by model. We also checked the models using a fixed threshold value of 10 out of 100 for the cumulative output. MaxEnt provides a convenient interpretation for the output of cumulative probabilities, where the expected omission rate for localities of the species is equal to the threshold employed. For example, an ideal model and a threshold of 10 would be expected to yield approximately 10% omission in an independent, unbiased sample of localities of the species. Hence, use of the fixed threshold of 10 is expected to lead to omission levels of approximately 10%.

Using an independent dataset is the optimal method for evaluating model performance [48], [65]. We used 6 *N. lanceopes* and 58 *N. antarcticus* occurrence records in the Ross Sea area extracted from the Ocean Biogeographic Information System (OBIS, www.iobis.org), the SCAR-Marine Biodiversity Information Network (SCAR-MarBIN, www.scarmarbin.be), and the published literature (Table S2). Records were filtered to remove duplicates (i.e. same co-ordinates or same records from different sources) and apparent geographic errors (i.e. co-ordinates plotting on land or in different regions) before combining them into a single data set for model verification using GIS. Probability of occurrence values, which ranged from 0 to 1, where 0 meant no probability of presence and 1 meant highest probability of presence at that particular location, were extracted from the average of all bootstrap models on each data set using the “Extract Values to Point” function of Spatial Analyst in ArcGIS. We evaluated model accuracy with the independent dataset by seeing how successfully the model predicted the species’ potential distribution outside its sampled distribution using six model evaluation metrics (each measured on a scale of 0–1), namely: Percent Correct Classification (PCC, overall accuracy); Sensitivity (the proportion of actual presences that are accurately predicted); Specificity (the proportion of actual absences that are accurately predicted); False Positive Rate; False Negative Rate, and True Skill Statistics (TSS, correct classification rate in relation to false positive rate) (see [66], Chapter 9).

## Results

### Sampled diversity and distribution

**Figure 3:**
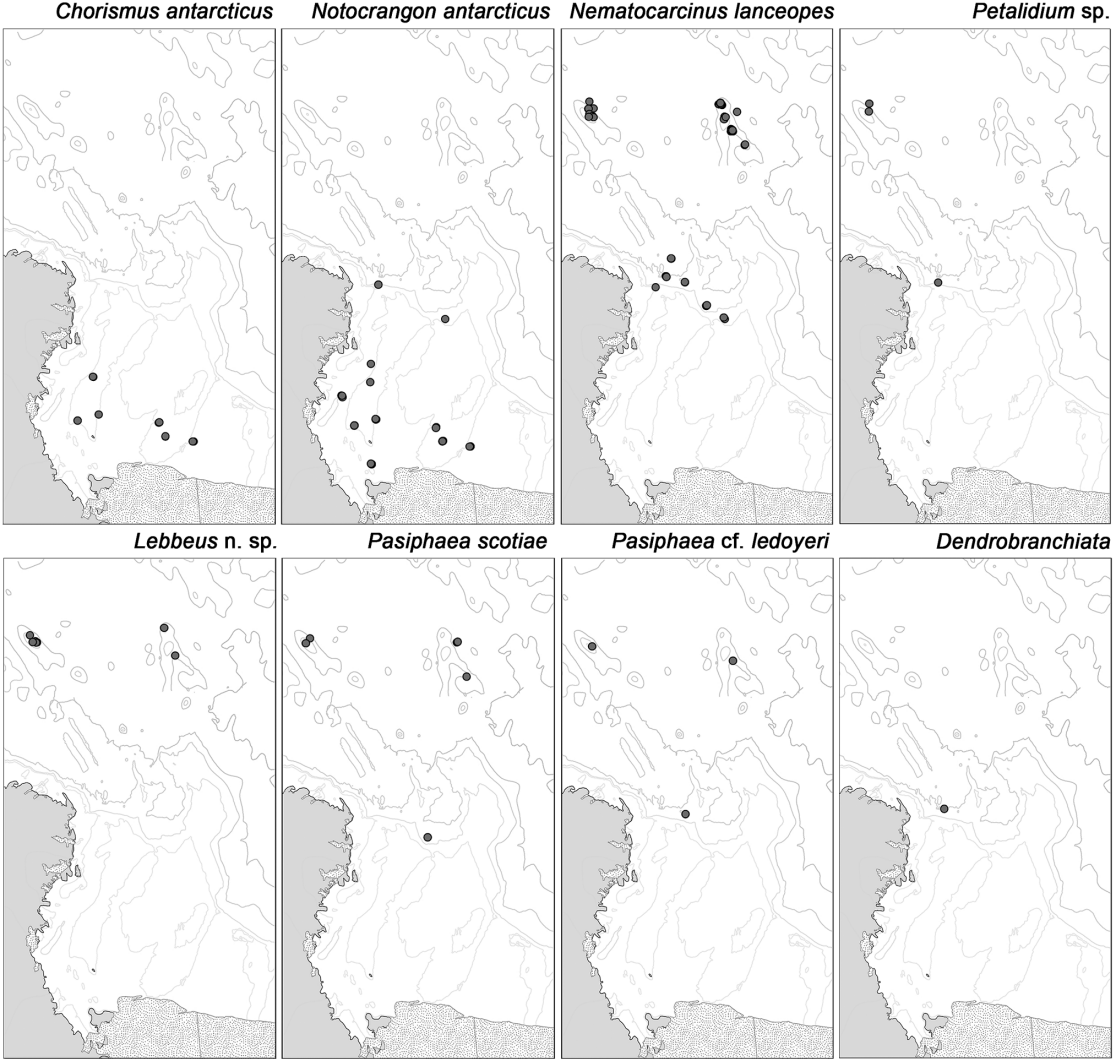
Spatial distribution of shrimp species sampled during NZ IPY-CAML voyage TAN0802 in the Ross Sea region.

**Figure 4:**
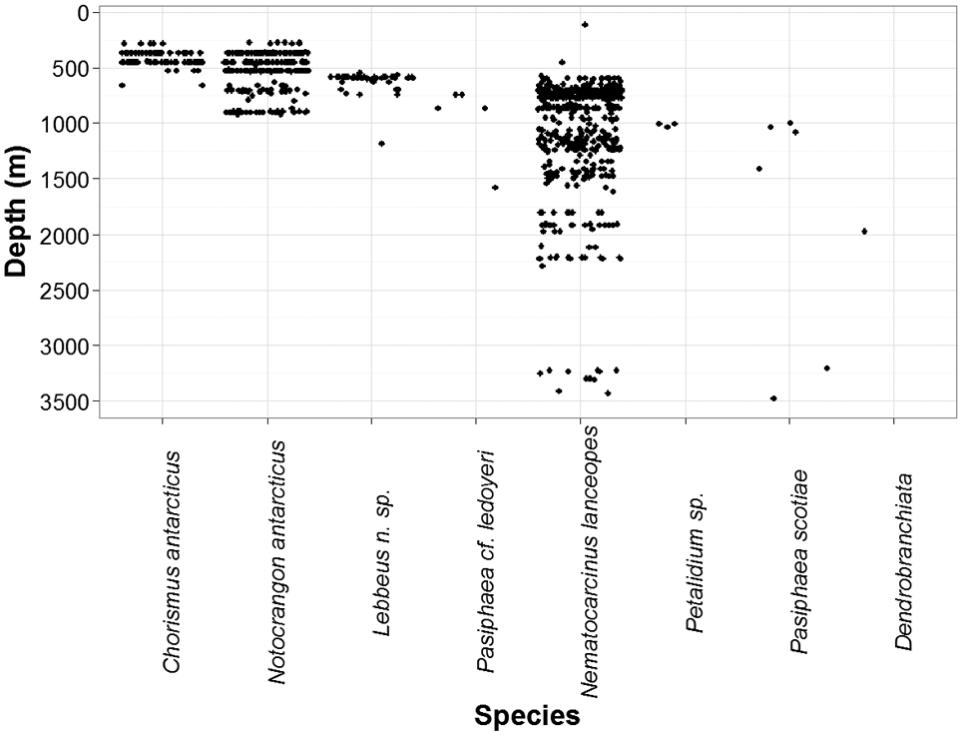
Depth ranges of sampled shrimp species during NZ IPY-CAML voyage TAN0802 in the Ross Sea Region.

In total, 921 shrimp specimens (91 preserved) were collected and 1249 individuals observed in video transects across 24 different sites (Table 1). Eight species were identified; *Chorismus antarcticus* (Pfeffer, 1887)*; Notocrangon antarcticus* (Pfeffer, 1887)*; Nematocarcinus lanceopes* (Bate, 1888)*; Pasiphaea scotiae* (Stebbing, 1914); *Pasiphaea* cf. *ledoyeri* (Hayashi, 2006); *Petalidium* sp.; an unidentified damaged specimen of the suborder *Dendrobranchiata*; and a new species of *Lebbeus* (S. Ahyong, unpublished data). *Chorismus antarcticus* and *Notocrangon antarcticus* were found only on the continental shelf in depths shallower than 1000 m. *Chorismus antarcticus* was largely restricted to depths shallower than 700 m, whereas *N. antarcticus* was found down to ca 1000 m at sites out to the edge of the continental slope. None of the other species were found on the continental shelf or at depths shallower than 450 m. *Nematocarcinus lanceopes, Petalidium* sp., *Pasiphaea* cf. *ledoyeri and Pasiphaea scotiae* were found on the continental slope and northern seamounts, but only *N. lanceopes* and *P. scotiae* were found at abyssal depths. *Dendrobranchiata* was found only at one site on the continental slope, and *Lebbeus* n. sp. was found only on the northern seamounts (Table 1 & Figure 3). *Notocrangon antarcticus* was the most frequently recorded species on the continental shelf (440 individuals, depth range 269–930 m) and *N. lanceopes* was the most frequently recorded species elsewhere (1554 individuals, depth range 570–3433 m) (Figure 4). Larvae of *N. lanceopes* were also recorded from MOCNESS [67] samples on the slope and seamounts (4 individuals, 110–800 m). The distributions of these two species overlapped at one site (D27) on the northern continental slope (Table 1) (Figure 3).

### Modelled distributions

**Figure 5:**
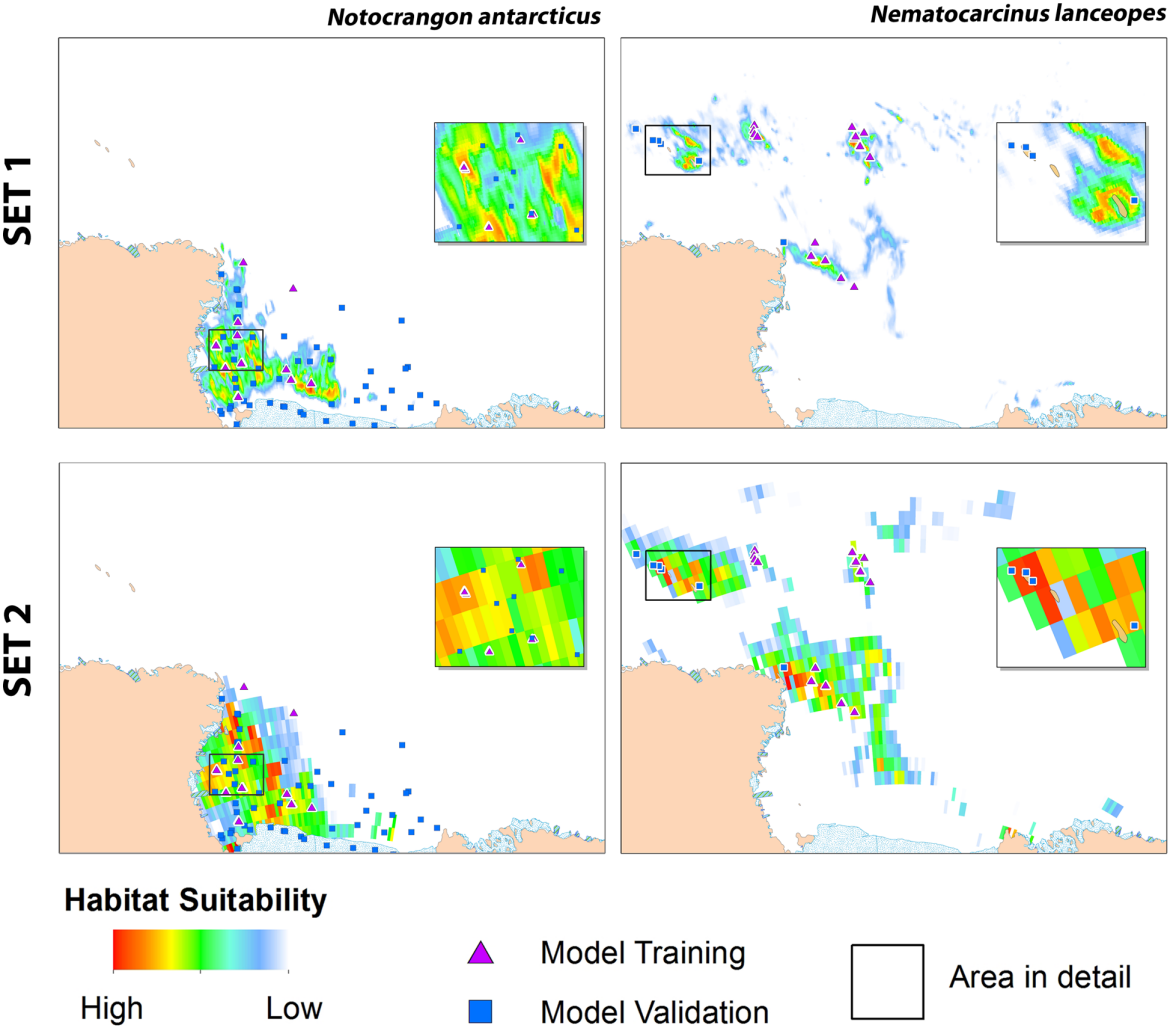
MaxEnt habitat suitability maps for *N. lanceopes* and *N. antarcticus* using two different resolutions of environmental data (SET 1, fine; and SET 2, coarse) in the Ross Sea region, showing predicted areas having values above low presence threshold value (LPT, see **Table 4**).

A total of 281 *N. antarcticus* and 909 *N. lanceopes* occurrence records were available from the TAN0802 cruise, including both physical specimens and records from video transects. When duplicate presence records within each grid cell were excluded there were 22 and 41 presence records at the fine spatial resolution (SET 1) for *N. antarcticus and N. lanc*eopes, respectively, and 12 and 17 records for the two species, respectively, at the coarser resolution (SET 2) (Table 4). For both *N. antarcticus and N. Lanceopes*, the extent of predicted suitable habitat was greater in the coarser spatial resolution model (SET 2) and less in the finer model (SET 1). There were also differences in the locations of highest probability of occurrence values between SET 1 and SET 2 models. This was particularly noticeable for *N. antarcticus,* for which the coarser resolution SET 2 models show wider distribution of suitable habitat across northern and western areas of the continental shelf than do the finer resolution SET 1 models (Figure 5). Both of the modelled distributions indicated geographic separation of the two species at the shelf break (Figure 5). The predicted distribution for *N. antarcticus* was restricted to the Ross Sea continental shelf, whereas suitable habitat for *N. lanceopes* was predicted to occur on the continental slope, Scott and Admiralty seamounts, and around the Balleny Islands, with lower probability of occurrence on the abyssal plain near these features (Figure 5).

**Table 4:** Results of model performance evaluation using different validation methods.

	*Notocrangon antarcticus*		*Nematocarcinus lanceopes*	
*Records*	SET 1	SET 2	SET 1	SET 2
**Training**	17	9	31	13
**Testing**	5	3	10	4
**Independent**	58	58	6	6
***AUC (Area Under Curve)***				
Training AUC	0.988	0.970	0.993	0.975
Test AUC	0.963	0.963	0.983	0.960
Training Gain	2.836	2.095	3.952	1.563
Test Gain	1.215	2.313	3.930	2.057
***Threshold***				
Low Presence Threshold (LPT)	0.168	0.431	0.031	0.432
P-Values for LPT	0.001	0.005	<0.001	0.001
10^Th^ percentile Threshold	0.291	0.431	0.141	0.484
***Omission Rate***				
Intrinsic	0	0	0	0
Extrinsic	0.09	0.07	0.05	0.07
***Independent Records***				
Maximum probability of presence (%)	64.66	74.40	59.56	86.19
Mean probability of presence (%)	24.03	46.74	19.60	80.92
Minimum probability of presence (%)	0.02	0.11	1.17	65.23
Standard deviation	0.11	0.18	0.08	0.21
Confidence Interval (95%)	0.03	0.05	0.06	0.17
Percent correct classification (PCC)	0.62	0.60	0.86	0.93
Sensitivity	0.52	0.66	0.67	1.00
Specificity	0.76	0.51	0.87	0.92
False positive rate	0.24	0.49	0.13	0.08
False negative rate	0.48	0.34	0.33	0
True Skill Statistics (TSS)	0.27	0.17	0.54	0.92

### Model evaluation

AUC values for both models were high (>0.9) and significantly different from a random prediction (Wilcoxon rank-sum test, *p*<0.01) (Table 4). High test gain (all values>1), indicated that <0.1% of the withheld test presences were misclassified. Intrinsic omission rates for all models were zero and extrinsic omission rates were <0.1, indicating acceptable model performance [29].

LPT and 10^th^ percentile presence threshold values were lowest at the finer spatial scale of SET 1 for both species; 0.168 and 0.291 for *N. antarcticus,* and 0.031 and 0.141 for *N. lanceopes,* respectively. Corresponding values using the coarser spatial scale in SET 2 were higher (0.431 and 0.431 for *N.* antarcticus, and 0.432 and 0.438 for *N.* lanceopes, Table 4). Because LPT is considered more suitable than the 10^th^ percentile in cases where presence records have been collected in a short period of time and with high spatial accuracy [68] as in the present study, we used the LPT values as the suitability cut-off value for model validation using independent records.

For both species, mean probability of independent location records plotting within the predicted habitat suitability area was highest at the coarse spatial scale (SET 2, mean ± SD; 46.7±0.19% and 80.9±0.21% for *N. antarcticus and N. Lanceopes*, respectively) and somewhat lower at the finer spatial scale (SET 1, 24.03±0.11% and 19.6±0.08%, respectively) (Table 4). Models of *N. lanceopes* had the highest accuracy based on the independent record evaluation metrics; in particular, PCC scores of 0.86 and 0.93 and TSS scores of 0.54 and 0.92 for SET 1 and SET 2 models, respectively. Corresponding values for *N. antarcticus* models were lower, at 0.62 and 0.60 for PCC and 0.27 and 0.17 for TSS, respectively.

### Environmental variables

Temperature and depth were correlated with each other in SET 1 (r = 0.75), and salinity and slope were correlated with each other in SET 2 (r = 0.88) (Table S1). MaxEnt has robust mechanisms integrated in the algorithm to deal with interactions of correlated variables [49], [69] so we did not exclude any variables from our variable pool.

**Figure 6:**
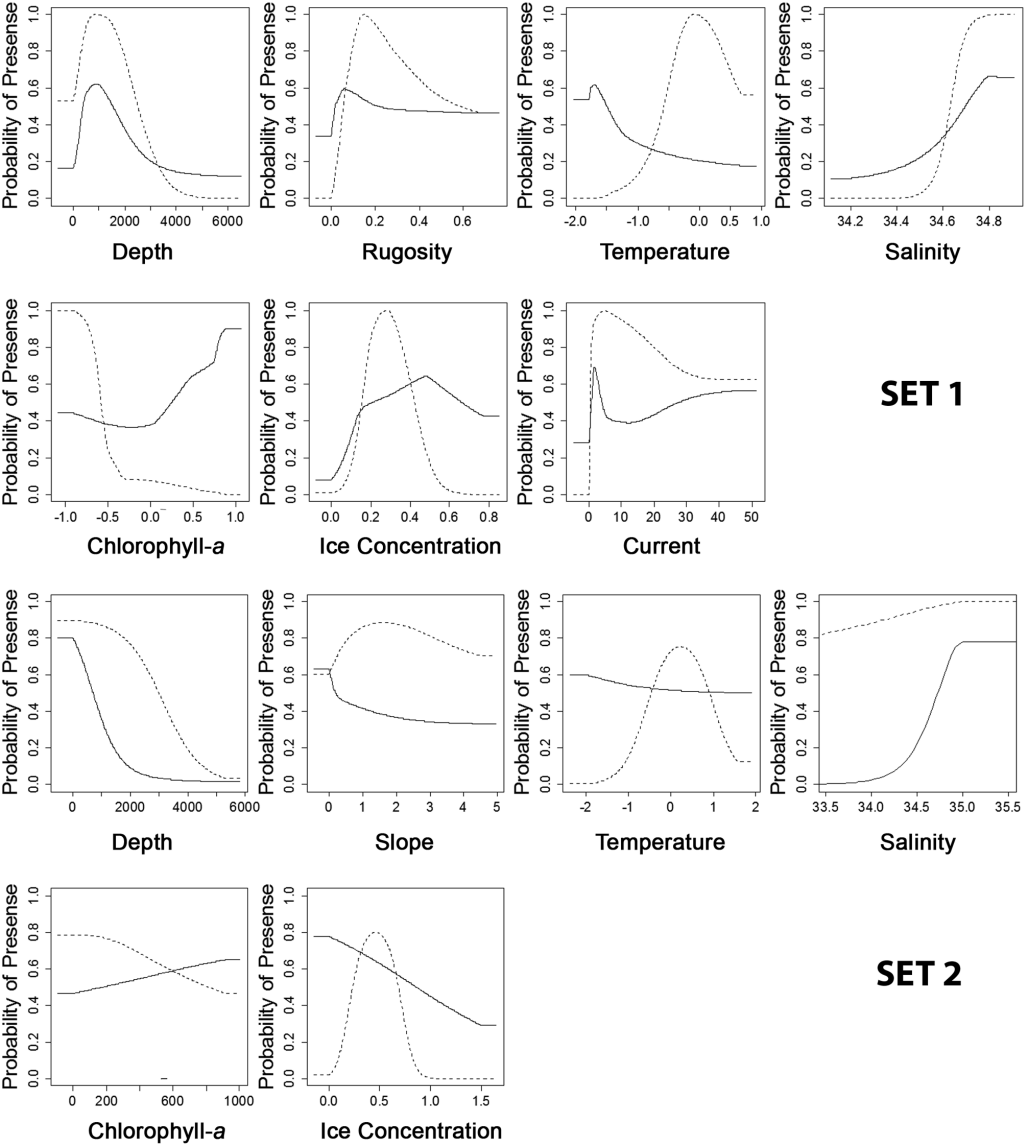
Response curves of environmental variables at two different spatial resolutions (SET 1 and SET 2) in MaxEnt models for *N. antarcticus* (solid line) *and N. lanceopes* (dotted line), showing how each variable affected model prediction performance.

MaxEnt model response curves show how the logistic prediction changed across the sampled range of each environmental variable, while keeping other variables at their average value (Figure 6). Each of these response curves represents a separate MaxEnt model created using only the named variable. The principal differences in environmental envelopes between the models of each species were in temperature range, chlorophyll-*a*, and ice concentration (Figure 6). The response curves indicated that *N. antarcticus* was likely to be found in lower seabed rugosity and slope areas that had colder waters with higher chlorophyll-*a* concentrations and lower ice concentrations than *N. lanceopes.*

In fine scale models of *N. antarcticus* using SET 1 variables, temperature, chlorophyll-*a* concentration, and depth had the highest contributions to the models, whereas using the coarse-scale SET 2 variables highest contributions were from depth, salinity, and chlorophyll-*a* concentration (Table 5). In the fine-scale SET 1 *N. lanceopes* models, highest variable contributions were from ice concentration, seabed rugosity, and depth, whereas in the coarse-scale SET 2 model, highest variable contributions were from depth, ice concentration, and temperature (Table 5). The maximum contribution of an individual variable to any model was 46.67% (depth, SET 2, for *N. antarcticus*). Jack-knife analyses of model gains, and test AUC scores for models generated with a single variable indicated that the same variables listed above were the top predictors regardless of covariation.

**Table 5:** Influence of environmental variables on the models generated using two datasets (SET1 and SET 2) for (a) *Notocrangon antarcticus* and (b) *Nematocarcinus lanceopes.*

(a) Notocrangon antarcticus	Contribution (%)		Jack-knife (Training gain)		Test AUC (Single variable)	
Variable	SET 1	SET 2	SET 1	SET 2	SET 1	SET 2
Depth	**9.22**	**46.67**	**1.607** !	0.812*	**0.924**	0.770
Rugosity	1.44	-	0.085	-	0.568	-
Slope	-	10.35	-	0.080	-	0.345
Ice Concentration	7.89	3.63	0.396*	0.135	0.735	0.692
Temperature	**45.24**	13.08	**1.436**	**0.976**	**0.923**	**0.820**
Salinity	1.10	**21.80**	0.894	**1.210**	0.898	**0.916**
Chlorophyll-a	**27.66**	**14.69**	**1.415**	**1.260** !	**0.911**	**0.941**
Bottom Current	7.45	-	0.167	-	0.648	-
**(b) Nematocarcinus lanceopes**	**Contribution (%)**		**Jack-knife (Training gain)**		**Test AUC (Single variable)**	
**Variable**	**SET 1**	**SET 2**	**SET 1**	**SET 2**	**SET 1**	**SET 2**
Depth	**15.67**	**29.55**	**1.250**	**0.496**	**0.881**	**0.806**
Rugosity	**29.14**	-	**1.290** !	-	**0.885**	-
Slope	-	10.35	-	**0.391**	-	**0.830**
Ice Concentration	**32.17**	**26.72**	**1.043** *	**0.922** ! *	**0.883**	**0.897**
Temperature	11.89	**25.53**	0.423	0.164	0.801	0.592
Salinity	7.61	6.58	0.215	0.142	0.699	0.627
Chlorophyll-a	5.51	1.28	0.178	0.017	0.692	0.558
Bottom Current	3.34	-	0.167	-	0.601	-

The top three environmental variables in terms of relative contributions are highlighted in **bold** for each species. Higher values for the regularised training gain of the jack-knife test indicated greater contribution to the model for a variable (these values were not directly comparable between the different species).

*indicates the variable that reduced the gain the most when omitted and therefore contained the most information that was not present in other variables

! **Indicates the variable with the highest gain when used in isolation and had the most useful information by itself.**

## Discussion

### Diversity and distribution

The NZ IPY-CAML survey has extended the number of known sites with species-level records of deep-sea shrimps in the Ross Sea and provided the first such records from seamounts and abyssal regions in the north of the region (Table S2, Figure 3 and Figure 4). These new observations have enabled us to re-evaluate known shrimp diversity and distribution in the Ross Sea region. Shrimps occurred throughout the region, with *N. antarcticus* being the most abundant species on the continental shelf, and *N. lanceopes* on the continental slope and seamounts to the north. *Notocrangon antarcticus* and *Chorismus antarcticus* occurred only on the shelf, whereas the five other species were only recorded off-shelf. These distributions reaffirm previous findings [1], [10], [11], [54], [70], [71], [72], [73], [74], [75]. However, previous surveys [76], [77], [78] did not find *N. lanceopes, Petalidium* sp. and *Dendrobranchiata* in the Ross Sea region, although a 2004 survey (NIWA unpublished data, [21]) found *N. lanceopes* in six locations at north western Ross Sea around slopes near Cape Adare and Balleny Islands (Table S2). Our results also show distinct depth zonation of *C. antarcticus, N. antarcticus,* and *N. lanceopes*, with a broad overlap between *C. antarcticus* and *N. antarcticus* in shelf regions, and between *N. antarcticus* and *N. lanceopes* on the upper slope; *N. lanceopes* being widely distributed in depths greater than 1000 m but less frequent in depth shallower than this (Figure 4). The new records of *N. lanceopes* and *Pasiphaea* spp. on seamounts north of the Ross Sea show that their distributions are more widespread than previously reported.

### Modelled distributions

Although the present data increase the number of records of the shrimps in the Ross Sea region considerably, the available data remain insufficient to map their distributions with confidence. Therefore, we used species distribution models to predict the geographic distribution of the two most common shrimps, *N. antarcticus and N. lanceopes,* based on their occurrences at 23 different locations in the Ross Sea region. This study is also the first in the marine environment to assess of the effect on species distribution model performance of using different environmental datasets at different spatial resolutions.

For all MaxEnt models of the predicted habitat suitability for both *N. antarcticus* and *N. lanceopes*, independent validation records plotted into areas with predicted maximum probability of presence between 59–86%, and all models had high AUC scores supported by high training gain and low omission rates, regardless of environmental dataset resolution. The AUC value tends to increase when the selected background area is larger than the species observed presence area [48], [53]. Thus, inclusion of other validation metrics is required for a thorough evaluation of model performance, particularly when our modelled species are known to have restricted distribution ranges (*N. antarcticus* in the shelf and *N. lanceopes* off-shelf) in a large geographic area. These results suggest that any of the modelled predictions are likely to be useful indications of distributions for these species, regardless of the spatial resolution of the underlying environmental data [29], [79], [80]. However, there was appreciable variation between outputs of the different models (Figure 5, Table 4) and it is important both to understand which environmental variables are influencing the models, and to consider factors that might underlie the differences between the models.

A recent study that modelled the distributions of *N. antarcticus and N. lanceopes* over the entire Southern Ocean using MaxEnt showed depth, ice concentration and salinity to have the highest explanatory power for models of *N. antarcticus,* while *N. lanceopes* distribution was better explained by depth, ice concentration and temperature [54]. In our study, at the scale of the Ross Sea region, depth, temperature, chlorophyll-*a* concentration, and salinity had highest explanatory power for *N. antarcticus*, whereas for *N. lanceopes*, ice concentration, depth, seabed rugosity, and temperature contributed most to the models. Given the spatial separation of these two species between the extreme high-Antarctic environment of *N. antarcticus* on the Ross Sea shelf and the more moderate oceanic environment of *N. lanceopes* beyond the shelf-break front, it is perhaps not surprising that these variables should contribute most to the models. Depth and seawater temperature are obvious distinctions between the two environments, shelf habitats being characterised by temperatures <0°C and depths <1000 m whereas beyond the shelf break temperatures are always >0°C and depths, other than on the seamounts, are >1000 m. However, the influence of the Ross Sea polynya also causes strong distinctions in ice concentration, salinity, and chlorophyll-*a* concentration between the environments of the two species (Figure 2). Seabed slope and rugosity are also important influences on benthic faunal distributions in the deep sea, e.g. by influencing food supply via current flow amplification [81]. Their influence in models, however, is likely to be strongly influenced by the spatial scales at which they are calculated. In our regional-scale models, the continental shelf break and slope, and the northern seamounts, are areas with high computed values for both slope and rugosity which contrast strongly with the comparatively uniform morphology of continental shelf and abyssal environments.

Because the steepest gradients in several potentially important variables coincide at the Ross Sea shelf break (depth, temperature, slope/rugosity, ice concentration, Chlorophyll-*a* concentration), determining which of these variables are most ecologically important to the realised distributions of the two species is problematic. Adaptation to cold has been postulated as the primary reason why Antarctic shrimps are capable of living at the extremely low temperatures of the continental shelf where other decapod taxa are absent [82], and as an explanation of why they were able to re-colonize high southern latitudes after past glaciation cycles [11], [82], [83]. That only two of the eight species identified here have distributions on the continental shelf, and that there is strong demarcation between species’ ranges at the shelf break, suggests either that such adaptation is species-specific or that factors other than physiological adaptation to low temperature *per se* have a stronger influence on realised distributions.

Physiological studies have suggested that many Antarctic benthic invertebrates on the continental shelf are highly stenothermal, and thus have limited capacity to withstand future environmental warming [84]. If the shrimp species studied here were currently range-limited by temperature, predicted warming might be expected to result in southward range shifts of those species currently found only in warmer waters north of the shelf break front (e.g. *N. lanceopes*, Figure 3). Conversely, for the two species with shelf-only distributions (*C. antarcticus* and *N. antarcticus*) at present, the only potential range shift would be southward into the region currently covered by the Ross Ice Shelf.

In addition to the suite of environmental variables used in species distribution modelling, three other factors were likely to affect the final outputs of the models and how well individual models rated in evaluation metrics. First, the number of independent records used to validate models can influence the test statistics [58]. In the present study, only 6 independent presence records were available to validate the *N. lanceopes* models, compared to 58 records for *N. antarcticus,* and it is likely that this will have had some effect on their respective validation metrics. Second, validation using independent records assumes geographic accuracy of the independent records; i.e., that the position data associated with these records are both accurate and precise. The accuracy of records derived from biodiversity databases can be uncertain, however [85], [86], and in the present study none of the records used for independent validation had spatial accuracy information associated with them. Therefore, it is possible that some of the independent records that plotted outside predicted areas of suitable habitat here might be as a consequence of such inaccuracies. Finally, the spatial resolution of the environmental datasets used in the models clearly influenced the predictions of the resulting models; this is discussed in more detail below.

### Effects of spatial resolution

Guisan *et al.* [32] suggested model performance depends more on the type of species, scale of the study area and modelling techniques than the spatial resolution of the used dataset. Although the four model validation techniques used here all suggested that the models in this study were useful predictions of potential distribution for the two shrimp species, there were some noticeable differences between models generated with datasets of different spatial resolutions. Models using the finer spatial scale dataset (SET 1) predicted areas of suitable habitat that closely matched the distributions of the observation records. By contrast, predicted areas with the coarser resolution dataset (SET 2), were broader. This is because that a decrease in the dataset resolution increases the size of individual grid cells and thus increases the probability that a given sample point will fall within areas of predicted suitable habitat. This was reflected during independent model evaluation, when mean probability of presence values were higher in coarse resolution datasets than finer resolution ones. Thus, finer resolution environmental data will tend to predict more restricted areas of occurrence, whereas coarse resolution data will predict wider potential biogeographic range, at least when using the default settings in MaxEnt. In addition to the influence of spatial resolution, it is also relevant here that the fine-scale data in SET 1 were developed more recently than those of SET 2 and were based on more extensive and detailed data from the most up-to-date observational and modelling sources [37]. While comparisons show that most layers are very similar between the two datasets (Figure 2), there are obvious differences in the summaries for Chlorophyll-*a* concentration that might be expected to have some influence on model results. Our results agree with the findings of terrestrial studies where model performance was not significantly affected by the coarsening of spatial resolution [31]. However, we found that the relative importance of environmental variables in predicting a species distribution varies with spatial resolution of dataset.

The most appropriate spatial resolution for modelling a species’ distribution will differ depending on that species’ ecological characteristics [87], [88], the amount and spatial accuracy of sample data available [53], [89], and the purpose of the modelling exercise. In this study, the relative importance of the environmental variables in explaining the species’ distributions differed depending on spatial resolution of the environmental data (Table 5), indicating that changing spatial resolution can influence the perceived importance of environmental variables. Environmental variables that characteristically change rapidly over short distances (e.g., in this case, depth, temperature, and ice concentration at the shelf break) are likely to have more influence in the finer resolution models than variables having more gradual rates of change over the study region (e.g., salinity). More fundamentally, models using coarser resolution data layers for SDM will not identify fine-scale variations in habitat suitability. This might have a strong effect in relation to the ecology of the modelled species as well. If relatively fine-scale topographic features (e.g. seamounts, canyon walls), are important habitat for a species and such features are appreciably smaller than the grid scale of the model, they will not be represented in the environmental data and thus will not be predicted in SDM predicted distributions. It is important, therefore, that the spatial resolution of species distribution models should be appropriate to the purpose of the modelling exercise.

Selection of a particular resolution (i.e. coarser or finer), for a species distribution modelling exercise in a practical application such as protected area design would depend on the specific management aim and whether or not decisions were to be based solely on the available data [90]. If the management aim is broad, for example, aiming to identify the best strategy for conservation of a poorly-sampled species with uncertain distribution, then using coarser resolution datasets would rapidly delineate regions of potentially suitable habitat with sufficient detail for decision-making purposes and be computationally less demanding. However, predicting core habitat areas of a species with well-understood environmental niche requirements will be more accurate with finer spatial resolution data.

## Supporting Information

Table S1 Pearson correlation matrix of environmental variables. Variables with high correlation highlighted in **bold**.
(DOC)

Table S2 Independent location records used for model validation.
(XLS)
