# Supplementary material for: Diversity and Distribution of Deep-Sea Shrimps in the Ross Sea Region of Antarctica
Source: PLoS One. 2014 Jul 22;9(7):e103195. doi: 10.1371/journal.pone.0103195 (PMC4106907; doi:10.1371/journal.pone.0103195)
Supplement: Table S1 — Pearson correlation matrix of environmental variables. Variables with high correlation highlighted in bold. (DOC) [file pone.0103195.s001.doc]

# Diversity and distribution of deep-sea shrimps in the Ross Sea region of Antarctica

**Zeenatul Basher^1*^, David A. Bowden^2^and Mark J. Costello^1^**

^1^Institute of Marine Science, The University of Auckland, Auckland, New Zealand,
^2^Coasts and Oceans Centre, National Institute of Water and Atmospheric Research (NIWA), Wellington, New Zealand

**Supporting Information**

**Table S1:** Pearson correlation matrix of environmental variables. Variables with high correlation highlighted in **bold**.

| SET 1 | ***Depth*** | ***Rugosity*** | ***Temperature**** | ***Salinity**** | ***Chlorophyll-a*** | ***Ice Concentration*** | ***Current**** |
| --- | --- | --- | --- | --- | --- | --- | --- |
| Depth | - |  |  |  |  |  |  |
| Rugosity | -0.307 | - |  |  |  |  |  |
| Temperature* | **0.751** | -0.108 | - |  |  |  |  |
| Salinity* | 0.346 | -0.015 | 0.195 | - |  |  |  |
| Chlorophyll-*a* | -0.381 | 0.021 | -0.386 | 0.111 | - |  |  |
| Ice Concentration | -0.228 | -0.029 | -0.305 | -0.237 | 0.344 | - |  |
| Current* | -0.125 | -0.028 | -0.083 | 0.016 | 0.108 | 0.070 | - |

| **SET 2** | *Depth* | *Slope* | *Temperature* ******* | *Salinity** | *Chlorophyll-a* | *Ice Concentration* |
| --- | --- | --- | --- | --- | --- | --- |
| Depth | - |  |  |  |  |  |
| Slope | 0.495 | - |  |  |  |  |
| *Temperature* ***** | 0.580 | 0.037 | - |  |  |  |
| Salinity ***** | 0.548 | **0.879** | 0.018 | - |  |  |
| Chlorophyll-*a* | -0.114 | 0.464 | -0.384 | 0.529 | - |  |
| Ice Concentration | -0.174 | 0.318 | -0.550 | 0.391 | 0.305 | - |

*** Bottom layers**
